# News and Coming Events

**Published:** 1909-09-25

**Authors:** 


### News and Coming Events.

Dr. A. T. Theobalds has resigned her post of lady resident medical officer at the Halifax Poor Law Infirmary.

Dr. F. E. Wynne has been appointed medical officer of health to the Leigh Town Council at a salary of £400 per annum.

Mr. F. M. Milne, B.Sc., M.B., has been appointed Lecturer in Clinical Pathology, and Mr. Charles Kerr, M.B., C.M., Clinical Medical Tutor in the University of St. Andrews.

Dr. Quarry, medical superintendent, Lambeth Poor Law Infirmary, and medical officer of the workhouse, has been granted leave of absence for six weeks to enable him to take a sea voyage, which it is hoped will have the effect of fully restoring him to health.

Mr. F. Bowreman Jessett, F.R.C.S., has resigned the post of surgeon to the Royal General Dispensary, Bartholomew Close, E.C. Mr. Jessett succeeded the late Mr. Morrant Baker as surgeon to the Dispensary, and has held the post for 27 years.

The inaugural address of the session 1909-10 of the University of Bristol will be delivered in the large hall on Thursday evening, September 30, at 8 o'clock, by the Vice-Chancellor of the University (Sir Isambard Owen, M.D.), who will take as his subject "The Significance of a University."

The opening address of the winter session at the Postgraduate College, West London Hospital, Hammersmith Road, W., will be delivered in the College on Monday, October 11, at 5 o'clock p.m., by Professor Theodor Schott, M.D., of Nauheim. Previous to the address tea and coffee will be served at 4.15 p.m.

We have received from the National Food Reform Association, 178 St. Stephen's House, Westminster, a new and revised edition of "Hints Towards Diet Reform, with 24 Simple Recipes," which contains an artistic frontispiece representing the Houses of Parliament as seen from the new offices of the Association. A specimen copy, price 3d. post free, may be obtained on application to the Secretary.

At an inquest at Bristol last week on a man who had died of pneumonia it was stated that while the nurse was leaning over his bed he snatched a hatpin from her cap and stabbed himself with it. The pin penetrated the body to a depth of about three inches and punctured the heart. The nurse pulled out the pin immediately and sent for a doctor, who found the man in a dying condition. The doctor attributed death to pneumonia and the wound in the heart.

It is proposed to add a clause to the Anæsthetics Bill, which is to be reintroduced next sessions, placing restrictions on the administration of cocaine. The Bill proposes to prohibit all but registered medical practitioners and dentists from administering anæsthetics for operations except under certain conditions, and it also seeks to prohibit the giving of a death certificate in the case of anyone dying while under the influence of an anæsthetic.

### RETIREMENT OF PROFESSOR CLELAND.

In view of the retirement of Professor John Cleland, M.D., F.R.S., from the Chair of Anatomy, at the end of the present month, there has been set on foot, on the initiative of the Business Committee of the General Council of the University of Glasgow, a movement for making appropriate recognition of his long and distinguished services. A circular has been issued to the whole body of University graduates and to members of other learned bodies with which Professor Cleland is or has been connected, bearing the signatures of the Earl of Glasgow, the Lord Provost of Glasgow, Sir John Stirling Maxwell, Bart., Sir James Fleming, Professor John Glaister, M.D., Sir Hector C. Cameron, M.D., the Rev. Dr. John Smith, and Principal J. Yule Mackay. In the circular it is stated that the form of recognition will, to a large extent, depend on the amount subscribed, but it is thought that it might fitly include the provision of some fund for the advancement of anatomical and anthropological science, and the presentation to the University of his portrait or bust by an eminent artist. A very representative committee has been formed, and the preliminary list of subscriptions, annexed to the circular, shows that the movement has already met with a very appreciative response.

A Scottish medical correspondent has sent us particulars of the career of this distinguished scientist and teacher. Dr. Cleland is in his seventy-fourth year, and is a native of Perth, where his father practised medicine. His professional education was obtained chiefly in Edinburgh, where he was a pupil of the celebrated John Goodsir. He also obtained part of his training in Paris and in Germany. At an early period of his career he acted as demonstrator to Professor Allen Thomson in the University of Glasgow, and afterwards he became Professor of Anatomy and Physiology in Queen's College, Galway, from which, after an occupancy of fourteen years, he returned to succeed Professor Allen Thomson in Glasgow. Professor Cleland has during his thirty-two years' incumbency of the Chair passed many thousands of students through his hands, and has enjoyed a great reputation as a teacher and as one of the most outstanding figures in scientific circles.

Among the most important of Professor Cleland's writings are his thesis on the structure and mechanism of the gubernaculum, published in 1856, and his papers upon the development of the skull and cranial variations, which appeared in the Philosophical Transactions of the Royal Society of London in 1862 and 1870. He collected in a volume entitled "Memoirs and Memoranda in Anatomy" many of his contributions to the scientific press of the last forty years. In his text-book, published in association with Dr. Mackay in 1896, he included much of his original observations made in the course of his life's work. Some of Dr. Cleland's most interesting and important papers are those containing his speculations on the great problems of thought as they present themselves to the naturalist. These were also collected and published under the title of "Evolution, Expression, and Sensation." In the Darwinian controversy, when it was at its height, Professor Cleland took an active part. Of his other works, "The Physical Relations of Consciousness and the Seat of Sensation," originally published in 1870, is also of note. The building up of the splendid museum with which the University of Glasgow is now enriched was to Dr. Cleland a labour of love in which he never wearied. Professor Cleland has had many honours conferred on him. Possessed of the artistic gift, his lectures on the relationship of sculpture and painting have always been received with the utmost appreciation. He is also the author of a work in verse.

Those desiring to take part in the recognition are invited to communicate with Mr. Archibald Craig, LL.B., 149 West George Street, Glasgow, the Clerk of the University General Council, who is acting as Secretary and Treasurer.

The medical staff and lecturers of the Royal Dental Hospital of London, 32 Leicester Square, W.C., will hold a conversazione and distribution of prizes on Wednesday, October 20, at 8 o'clock p.m., at the Royal Institute Galleries, Princes Hall, Picadilly. The distribution of prizes will be made at 8.30 p.m. punctually by Sir Victor Horsley, F.R.C.S., F.R.S., and this will be followed by music, etc.

A reception of the members of the Child Study Society, London, will be held by the President, the Right Hon. the Earl of Stamford, on Thursday, October 7, at 8 p.m., at 90 Buckingham Palace Road, S.W. Short addresses on Child Study will be given by Miss Alice Ravenhill, C. W. Kimmins, D.Sc., M.A., and G. E. Shuttleworth, B.A., M.D. Morning dress will be worn. Tea, coffee and light refreshments will be served during the evening.

The winter session at Guy's Hospital Medical School will commence on October 1. The first meeting of the Pupils' Physical Society will be held on October 7, when Sir Hector Cameron will read a paper entitled "A Plea for the Treatment of Abscess by Lister's Antiseptic Method." Previous to the meeting a dinner of old students will be held in the Students' Club, when the chair will be taken by Mr. H. Cosmo Bonsor, the treasurer of the hospital.

At a special meeting of the governors and subscribers of the Birmingham and Midland Eye Hospital, held on September 16, Mr. Walter Wilkinson presiding, it was proposed and seconded that Law 7 should be altered to read as follows: "That donors and subscribers shall have the privilege of exchanging an in-patient ticket for six out-patient tickets; subscribers of two guineas and a donor of twenty guineas shall have the privilege of exchanging six of his or her own out-patient tickets for one in-patient ticket, on application to the secretary. In-patient tickets to continue in force fourteen days; out-patient tickets to continue in force six weeks. That no persons shall be admitted as a patient to the institution without a ticket except in cases of accident or sudden emergency." After a short discussion, Mr. Conway Lowe put forward an amendment, but, as this could not be discussed without a month's notice having been given, it was decided to put the original motion to the meeting. This was done, and the proposal was defeated by four votes to three.

In the House of Commons on September 16, Capt, Craig asked the Home Secretary whether he would consider the advisability of issuing regulations compelling manufacturers of medicines liable to patent-medicine duty to print on the label the full ingredients, as well as the diseases they purported to cure; and whether he would consider the advisability of increasing the size of the lettering of the Government stamp as a further precaution against ignorant people believing that the contents were guaranteed by the Government. Mr. Gladstone replied: "I am in communication with the Privy Council Office about the point raised in the first part of the question, but I am disposed to think that the matter may be one of sufficient importance for an inquiry by a Select Commission next session. As regards the second part of the question, I am informed that new designs for medicine stamps have recently been adopted, and the printing plates are being prepared by the engravers. In the new designs it will be more clearly indicated that the stamp does not imply any Government guarantee." Captain Craig: "Will the committee be appointed next session?" Mr. Gladstone said he thought it would be desirable. Mr. A. Lynch asked "whether in certain foreign countries it is not a matter of obligation to print the full description," to which Mr. Gladstone replied that this might be so.

Dr. J. R. Steinhaeuser has been reappointed medical officer of health for Lewis.

The jubilee of the National Hospital for the Paralysed and Epileptic (Albany Memorial) will be celebrated at the hospital on Saturday afternoon, October 9. H.R.H. the Princess of Wales will receive the Jubilee Purses at 3.15 o'clock. Cards of admission will be issued by the Secretary at the Hospital.

The Home Secretary has signified to the Council of the Royal College of Surgeons of England his decision to approve the by-laws regarding the admission of women to the examinations for the diplomas of the College. He has further expressed his willingness to sign the formal document which is to be submitted after the next meeting of the Council of the College on October 14. It is understood, however, that meanwhile it will be possible to complete the necessary formalities in time for women to enter for the examinations of the Royal College to be held in January next.

